# Healthy Snacks and Drinks for Toddlers: A Qualitative Study of Caregivers’ Understanding of Expert Recommendations and Perceived Barriers to Adherence

**DOI:** 10.3390/nu15041006

**Published:** 2023-02-17

**Authors:** Jennifer L. Harris, Maria J. Romo-Palafox, Haley Gershman, Inna Kagan, Valerie Duffy

**Affiliations:** 1 Rudd Center for Food Policy & Health, University of Connecticut, Hartford, CT 06103, USA; 2 Nutrition and Dietetics, Doisy College of Health Sciences, Saint Louis University, St. Louis, MO 63104, USA; 3 Department of Allied Health Sciences, College of Agriculture, Health and Natural Resources, University of Connecticut, Storrs, CT 06269, USA

**Keywords:** food parenting, toddlers, healthy snacking, sweet and salty snack, sugar-sweetened beverages, nutrition education

## Abstract

Background. Despite expert recommendations, most toddlers consume sugary drinks and more sweet and salty snack foods than fruits and vegetables as snacks. Studies have examined toddler caregivers’ reasons for providing sugary drinks, but few have examined the reasons for providing nutritionally poor snack foods. Methods. Researchers conducted focus groups in one low-income community to assess caregivers’ familiarity, understanding and attitudes regarding healthy drink and snack recommendations for toddlers. A convenience sample of 24 caregivers of toddlers (12–36 months) participated. Researchers conducted a descriptive analysis of the participants’ familiarity with recommendations and a thematic analysis of the barriers to adherence. Results. Most participants were familiar with recommendations, but many were surprised that some drinks and snack foods are not recommended, and most believed recommendations were not realistic. Common barriers to adhering to recommendations included beliefs about their child’s innate preferences, family modeling and others’ provision of drinks and snacks in and outside the home. Practical barriers included the higher cost and inconvenience of serving fruits and vegetables on-the-go. Conclusion. Similar barriers limited caregivers’ adherence to expert recommendations about healthy snacks and drinks for toddlers. Nutrition education interventions should provide practical strategies for addressing these barriers and enlist childcare and health providers to reinforce recommendations.

## 1. Introduction

Early feeding practices influence children’s long-term food preferences, eating habits and health outcomes [1]. In particular, the first 1000 days (from pregnancy through 2 years) is a critical time for developing preferences for nutritious foods and avoiding foods that are high in added sugar and salt. The transition from a milk-based diet to family diets during the toddler years provides a window of opportunity to shape healthy food and drink consumption habits [1,2]. In addition to main meals, snacks provide important nutrients for toddlers and a further opportunity to develop preferences for nutritious foods [1,3]. As a result, experts recommend serving fruits or vegetables at most snacking occasions while avoiding highly processed foods, including salty snacks and snacks with added sugars [1,4]. In 2019, a group of child health experts also published healthy drink guidelines, recommending that children younger than 5 years of age should drink plain water and cow’s milk, limit 100% juice and avoid all beverages with added sugar [5].

However, U.S. toddlers’ diets often deviate from these expert recommendations. Data from the 2016 Feeding Infants and Toddlers Study (FITS) indicate that, on a given day, 25% of 1-year-olds and 45% of 2- and 3-year-olds consume a sugar-sweetened beverage, primarily fruit-flavored drinks [6,7]. Nearly all toddlers consume a sweet snack, a sugar-sweetened beverage or dessert daily, and one-third consume salty snacks [6,7]. Moreover, more than 20% do not consume any fruit, and 40% do not consume any vegetables (excluding potatoes) [6,7]. An analysis of the snacking patterns of young children (2–5 years) using 2005–2012 NHANES data found that products in the “snacks and sweets” food category (e.g., cookies, crackers) were the leading contributors of the total energy (44%) and total fat (52%) consumed outside of main meals [8]. Moreover, sweetened foods (including bakery products, other desserts and candy) contributed 53% of the added sugars consumed as snacks, while sugary drinks (including soda and fruit drinks) contributed another 25% [8]. In total, foods and drinks consumed at snack times contributed 39% of young children’s total daily sugar intake (22 g) [8]. An analysis of earlier (2008) FITS data compared consumption by eating occasions and found that, at that time, fruits were the most common snack food for toddlers and preschoolers (12–48 months), consumed by 37% to 46% on a given day [9]. However, cookies and crackers also ranked among the top five foods consumed as snacks, and the consumption of chips and other salty snacks increased with age, from 11.5% of 12- to 24-month-olds to 18% of 36- to 47-month-olds [9].

Research for informing nutrition education and public health campaigns in order to reduce sugary drink consumption, often conducted with low-income and/or Hispanic caregivers, has examined barriers to following healthy drink recommendations for young children. Individual factors include the parent and family consumption of sugary drinks [10,11,12], perceptions of children’s beverage preferences [11,13,14] and giving in to children’s requests [11,15,16]. Commonly cited structural barriers include the ready availability of sugary drinks (such as in fast food restaurants and childcare), the greater convenience of unhealthy options, the marketing of unhealthy products directly to children [11,12] and the higher cost of healthy options [11,12,14]. Studies have also demonstrated limitations in caregivers’ understanding about healthy drink recommendations—primarily, beliefs that sweetened fruit drinks are not sugary drinks [13,17,18] and confusion due to conflicting messages from different expert sources, such as pediatricians and counselors for the Special Supplemental Nutrition Program for Women, Infants and Children (WIC) [10,19].

In contrast, few studies have examined caregivers’ understanding or attitudes regarding healthy snack recommendations for toddlers, including their perceptions and beliefs about the provision of sweet and salty snack foods and the reasons for the less frequent provision of fruits and vegetables as snacks. Some young child feeding studies provide potential explanations by identifying differences in nutrition quality by eating occasion. For example, one U.S. study found that toddlers consume more sweets, sweetened drinks and desserts when snacking away from home compared to snacking at home [20]. Another study of toddler eating habits in the home environment found that toddlers were more likely to consume fruits and vegetables when not moving around or watching TV and when eating at the table [21]. In research with preschoolers, snacks that were provided in some contexts (e.g., in social situations) were less nutritious than those offered in routine situations or to address hunger [22]. 

Other studies have found that caregivers also differ widely in their definitions of what constitutes a “snack”, which typically incorporates certain types of food, provided in smaller portions and between meals [23,24]. Another study with caregivers of infants (6 months) identified a common belief that snacks are sweet [25]. One qualitative study with Dutch parents found numerous value conflicts in parents’ decisions about what snacks to serve their children (2–7 years), including healthfulness versus convenience, judgments by other parents, saying “no” to child requests and less nutritious snacks provided by others [15]. However, U.S.-based studies have not specifically examined the reasons for the low adherence to expert recommendations about healthy snacks. 

The present qualitative study aimed to build upon research that has examined barriers to following expert recommendations for healthy drinks. Conducted with caregivers of toddlers (12–36 months), it also aimed to provide insights into caregivers’ reasons for providing nutritionally poor sweet and salty snack foods versus fruits and vegetables as snacks, including their knowledge and understanding of the recommendations and their experiences with providing different options to their young children. These findings will help identify potential approaches for assisting caregivers of toddlers in replacing highly processed sweet and salty snacks, as well as sugary drinks, with healthier options.

## 2. Materials and Methods

We conducted in-person focus groups with caregivers of toddlers living in one Connecticut community from March to June 2019. The discussion topics and stimuli presented in the groups addressed common toddler feeding practices that did not follow expert guidelines, including the frequent provision of sugar-sweetened drinks and nutrient-poor sweet and salty snacks, the infrequent provision of fruits and vegetables as snacks and behaviors not consistent with responsive feeding practices. These practices had been identified in an online survey conducted in the same community that utilized a convenience sample (*N* = 143) of low-income parents of toddlers at various child-focused locations [26]. The focus groups were designed to better understand these feeding practices through open and honest discussion regarding caregivers’ understanding of expert recommendations about toddler feeding and their own experiences in feeding their young children.

To facilitate the discussion, researchers and registered dietitians created concept sheets around topics identified in the online survey, including “What should my toddler drink”? and “What snacks should I give my toddler”? A third topic was also discussed: “How much should my toddler eat”? Although the results of this topic are not presented here, we note its inclusion to disclose the full context of the discussion. 

### 2.1. Participants and Recruitment

We held the focus groups in one low-income community with racial/ethnic diversity. The community includes 36% Hispanic, 34% non-Hispanic White and 24% non-Hispanic Black residents [27]; 22% of residents receive Supplemental Nutrition Assistance Program (SNAP) benefits, a U.S. government program that provides food purchase assistance to eligible households; and 50% or more of the children are eligible for free and reduced-price meals from the U.S. Department of Agriculture (USDA) school nutrition program [28]. To recruit participants, the researchers distributed flyers (in English and Spanish) at locations serving parents of young children, including family resource centers, WIC offices, libraries and early education centers. The research personnel also visited some of these locations and provided information about the study to potential participants (i.e., individuals who care for a toddler-age child) in person. The researchers, including a native Spanish-speaker, contacted interested parents by phone or email to describe the study, discuss the participation requirements and screen participants to ensure that they cared for a toddler (12–36 months old), made most decisions about what to feed that child and did not have a child who follows a special diet for medical reasons.

The researchers then scheduled interested and eligible participants for a focus group time and place based on the participant availability and location preference. Two groups were held at a WIC office and included only WIC participants. One group was conducted in Spanish. Childcare was provided at all locations, if required. The participants received a USD 20 retailer gift card for their time. The University’s Institutional Review Board approved all of the study procedures (protocol X19-009).

### 2.2. Procedures and Stimuli

The research team included the lead researcher and a post-doctoral researcher, a registered dietitian with a PhD in nutrition sciences and a native Spanish speaker, who both served as moderators. Two dietetics students coded the transcripts and assisted in the descriptive and thematic analyses. The lead researcher trained the research team members in qualitative methods. The focus groups met at a local WIC office, family resource center or public library. The groups lasted for approximately 90 min. Upon arriving at the focus group location, the participants read and signed an informed consent form in English or Spanish. One of the moderators, with assistance from the other moderator, led all groups.

The moderator informed the participants that the focus group would be audio-recorded and transcribed without any identifying information. The moderators used a discussion guide with semi-structured questions to facilitate the conversation. To begin, the moderator explained the protocol for the session and answered any questions. As an icebreaker, the participants introduced themselves and shared information about their background, their child’s age, if anyone else helps decide what to feed their child and any challenges they currently experience in obtaining food or feeding. 

**Figure A1:**
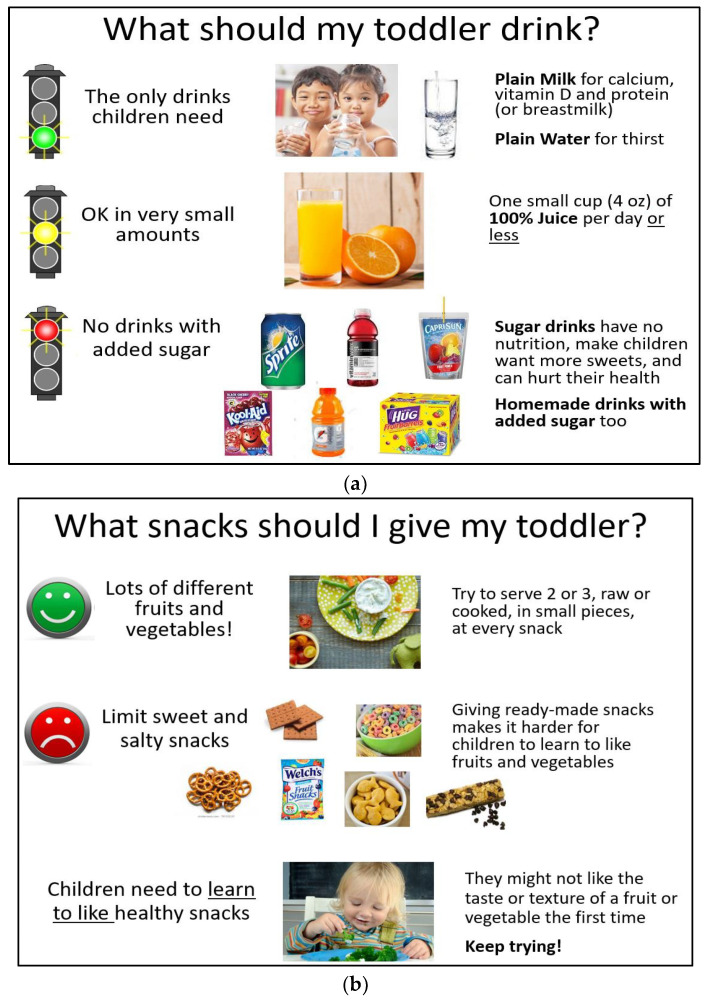
Stimuli presented in the focus groups: Expert recommendations for (**a**) drinks for toddlers; (**b**) snacks for toddlers.

**Figure A2:**
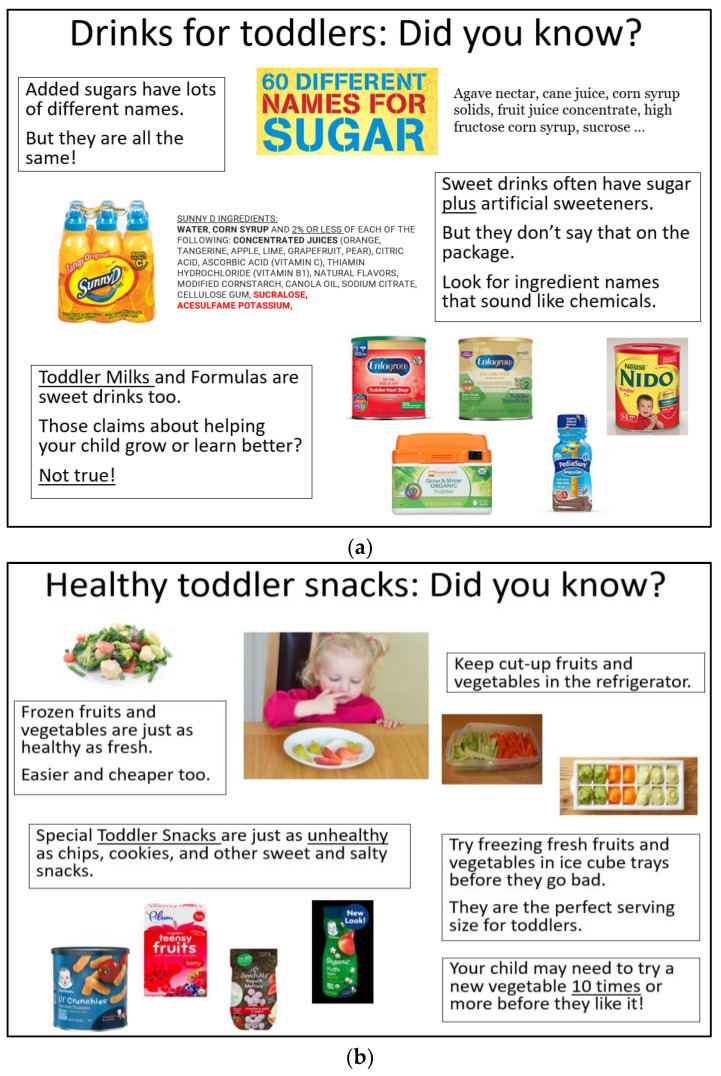
Stimuli presented in the focus groups: Additional information and tips for (**a**) drinks for toddlers; (**b**) snacks for toddlers.

Two concept sheets were created for each topic (see Appendix A). The first, “What should my toddler eat/drink”, (Figure A1) presented recommendations from nutrition and child feeding experts regarding healthy drinks (“The only drinks children need” (plain water and milk), “Ok in very small amounts” (100% juice) and “No drinks with added sugar”) and snacks (“Provide lots of different fruits and vegetables” and “Limit sweet and salty snacks”) for toddlers. The second concept sheet for each topic, “Did you know”, (Figure A2) included information that might be new to participants (e.g., “Special toddler snacks are just as unhealthy as chips and cookies”) and practical tips to help them follow the guidelines (e.g., “Try freezing fresh fruits and vegetables in ice cube trays before they go bad”). Each sheet was designed to be easy-to-read, with pictures illustrating the messages, including examples of popular branded products marketed for toddlers and/or young children. Research advisors who were experienced in conducting nutrition education with SNAP and WIC participants and/or had expertise in optimal child feeding and communications provided input in developing the concepts, approved the final concept sheets and advised the protocol development. The Spanish-speaking researcher translated the concept sheets for the Spanish-speaking group.

Following the icebreaker discussion, the moderator distributed copies of each concept sheet and discussed the topic before distributing the next concept sheet. For each topic, the moderator asked the participants how they felt about the recommendations, if they had heard them before, if they thought they were realistic and what might make them hard to follow. To wrap up, the participants shared their final thoughts, including any new information they had learned. Data saturation was achieved in groups six and seven. During these groups, the participants contributed no new information about barriers to following the recommendations, and the researchers determined that no additional groups were necessary.

### 2.3. Data Analysis

A professional service transcribed the audio recordings of the focus groups verbatim and translated the transcription of the Spanish-language group to English. The native Spanish-speaking moderator reviewed the translation of the Spanish-language group against the original audio recording for accuracy. Members of the research team first reviewed the transcripts and developed the codebook to (1) identify quotes that directly answered the moderator’s questions about whether caregivers had heard the expert recommendations before and whether they followed the recommendations and (2) identify caregiver quotes that elucidated potential barriers to serving healthier drinks or snacks and/or not serving sugary drinks or the highly processed sweet and salty snacks depicted. The codebook described potential barriers, including those identified in previous studies [10,11,12,13,14,15,16,17,18,19,20,21,23,24] and additional barriers identified in the initial review of the transcripts. The coders also classified all quotes according to whether they referenced drinks or snack foods.

Upon the development of the initial codebook, two coders first coded one focus group transcript by independently assigning caregiver quotes to codebook items using NVivo 12 (QSR International, Burlington, MA, USA, 2018). The lead researcher and coders met to discuss discrepancies and refine the codebook definitions until the research team reached a consensus. Both coders then coded the second focus group transcript and achieved 80% interrater reliability in assigning caregiver quotes to all items in the codebook [29]. The lead researcher met with the coders one more time to refine and merge codes in order to obtain a consensus on all of the remaining discrepancies and finalize the codebook. The coders then independently coded the remaining transcripts. 

After the coding was completed, the research team first examined the quotes that described the participants’ familiarity with the recommendations and whether they followed them. Using these quotes, they conducted a descriptive analysis of the caregivers’ awareness and adherence to the recommendations. Then, the lead researcher and one of the coders (a master’s student and registered dietitian) combined and classified codes about barriers into potential themes for a thematic analysis of the potential barriers to following expert recommendations [30]. These themes represented the underlying reasons why caregivers did not follow the recommendations for healthy drinks and snacks. All members of the research team reviewed and provided feedback on the results of this thematic analysis. 

After reviewing the themes identified in the initial thematic analysis and receiving external feedback from advisors, the researchers re-examined the quotes for meaningful patterns between themes and for similarities and differences between the perceptions of recommendations for drinks versus snacks. Quotes regarding food waste were recategorized as referencing either their child’s innate preferences or the cost of throwing away food their child would not eat. Some barriers examined in the codebook were mentioned fewer than 10 times across all groups (i.e., eating restrictions, celebrations/special occasions, marketing and culture) and were not included in the final thematic analysis. The final thematic analysis included three main themes, with respective sub-themes, to describe the underlying reasons why caregivers may not follow expert recommendations (see Table 1). 

Although the focus group discussions and thematic analysis focused on barriers that caregivers experienced in following expert recommendations, a few examples emerged during the coding when caregivers described positive influences and strategies that facilitated their child’s consumption of healthier drinks and snacks. These examples did not meet the minimum number of mentions to qualify as a sub-theme, but researchers noted them under the related sub-themes as potential strategies for addressing some common barriers.

## 3. Results

We conducted seven focus groups, including two groups at the WIC office and one group in Spanish. The groups included two to six participants each. The 24 participants included 20 mothers, 1 father and 3 other caregivers (2 grandmothers, 1 aunt). Seven participants self-identified as Black non-Hispanic, eight self-identified as Hispanic and two self-identified as Asian during the discussion. All participants cared for at least one toddler (12–36 months) and made decisions regarding what to feed them; most cared for more than one child. Six WIC participants attended the groups at the WIC office. 

### 3.1. Descriptive Analysis: Awareness and Adherence to Recommendations

Most participants indicated that they had heard the recommendations about healthy drinks for toddlers, including from pediatricians, WIC counselors, dentists and/or nutrition students working with the childcare centers. Some participants stated that they followed the guidelines, especially the recommendation to regularly provide water to their child: “*But most of the time it’s water or milk. And more water than milk. She* [my toddler] *drinks a lot of water”.* However, most indicated that they did give their child sugary drinks and/or more than the recommended amount of juice at times. In addition, many indicated that both the healthy drink and snack recommendations were not realistic. As one parent in a group held at the WIC office noted: “*It’s the recommendation we hear from everybody. Here* [WIC], *the pediatrician. Do I follow it? No.”* These discussions were especially robust among the WIC participants: *“This is not reality. I mean, not in my house. I’ve tried to do what WIC tells me to do. I do try”,* and *“Yeah, it is hard to be strict like that. I know there’s some parents that can do it, and I commend them. But I don’t know”.*

Similarly, nearly all participants had previously heard that toddlers should consume a variety of fruits and vegetables as snacks and that they should limit sweet and salty snacks. Many indicated that they regularly provided fruit to their child for snacks. In the words of one WIC participant, “*And I mean, we eat lots of fruit in our house. That’s the sweet that I want him to have”.* However, the participants frequently stated that serving a variety of different fruits and vegetables, especially vegetables, was unrealistic. In addition, the caregivers often served their child sweet and salty snacks that were included on the concept sheet list of non-recommended products, including those who suspected that the snacks they served were less healthy: *“But you have to be realistic and know that you’re going to have junk in your house or there’s going to be junk around. You can’t keep your kids off sugar”.*

### 3.2. Thematic Analysis: Barriers to Following Expert Recommendations

Despite the caregivers’ perceived familiarity with the expert recommendations for healthy drinks and snacks for toddlers, three main themes emerged as common barriers that made it difficult to follow the recommendations: (1) limitations in their understanding of the recommendations; (2) beliefs that their child prefers sugary drinks and/or sweet and salty snacks over healthier drinks or fruits and vegetables; and (3) practical considerations. Table 2 lists representative quotes to describe these themes and the sub-themes within each one. 

#### 3.2.1. Theme 1: Limitations in the Understanding of Expert Recommendations

One common sub-theme that emerged during the discussions about healthy drink and snack recommendations focused on misperceptions about some of the non-recommended sugary drinks and sweet and salty snacks that were included as examples on the concept sheets. Most caregivers understood that soda was a sugary drink that toddlers should not consume. However, some expressed surprise that other types of sweetened drinks, including fruit drinks, flavored water and sports drinks, were also considered to be sugary drinks that experts do not recommend for young children. Others were unsure whether some juices had added sugar and would be considered sugary drinks. Similarly, many caregivers were surprised about the products included as examples of sweet and salty snacks not recommended for toddlers, including goldfish crackers, graham crackers, pretzels, granola bars and baby food snacks. They frequently indicated that they had believed these snacks were healthy for toddlers. This confusion often appeared to arise when other people who are trusted provided these products to their child, such as in school and other child-oriented settings.

Another sub-theme focused on questioning the healthy drink recommendations, especially during the WIC groups. These questions arose when caregivers perceived that they had received inconsistent information from pediatricians and other trusted people. Some mentioned that WIC counselors’ advice conflicted with other trusted sources, especially concerning serving cow’s milk and the recommended amounts of milk, and that it was less flexible than advice from others. 

#### 3.2.2. Theme 2: Child’s Food Preferences

The most frequently cited barrier to following the expert recommendations for both drinks and snacks revolved around caregivers’ beliefs that their child would not consume healthier options, including plain water and a variety of fruits and vegetables, and/or would only consume non-recommended products. One common sub-theme described beliefs about their child’s preferences. In many cases, caregivers had heard that their child might need to try new vegetables several times before they would eat them, but concerns about food waste made that advice difficult to follow. Caregiver statements also often seemed to imply that their child’s preferences were innate characteristics and would not change. 

Two additional sub-themes focused on outside influences that are believed to affect their child’s food preferences: the modeling of unhealthy behaviors by family members and the provision of drinks and snacks that do not meet recommendations, including by other family members and others outside the home. Parents often described how their toddlers’ food preferences were affected when they saw others in their household, including older siblings and even the caregivers themselves, consuming sugary drinks and sweet and salty snacks. Some caregivers described trying to get their toddler to eat according to the recommendations while seeming to imply that it was too late for their older children. The participants also provided many examples of other family members, most often grandparents and fathers, giving their children sugary drinks or unhealthy snacks when caring for them, often in conflict with the participants’ wishes. They also expressed concerns about non-recommended drinks and snacks provided to their child in settings outside of the home, including in childcare. 

In both sub-themes, the caregivers expressed frustration that others’ behaviors undermined their own attempts to get their child to consume recommended drinks and snacks, but they felt unable to control those behaviors or believed that stopping them would require extraordinary effort. The caregivers’ statements also often implied that once their child tried sugary drinks and/or sweet and salty snacks, they would prefer those options over fruits and vegetables or plain water and milk and even refuse the healthier options altogether. 

In the discussion of outside influences on their child’s preferences, a few parents also described healthier practices by childcare providers, citing them as informing their own feeding practices. For example, one parent stated, “*And one thing I like with the preschool that my son goes to, there is no juice whatsoever. It’s just water and milk”.* Another commented about an older child, “*Now, my 4-year-old boy had started to eat veggies, but because I caught him, that he always eats them at school. And I was like, oh, so you always eat them at school, so you’re going to eat them here”.*

#### 3.2.3. Theme 3: Practical Considerations

This final theme focused primarily on barriers to serving fruits and vegetables for snacks, including sub-themes related to the cost, convenience and relative availability of sweet and salty snacks versus non-recommended options. The participants often mentioned the higher cost of fruits and vegetables and the lower cost of non-recommended options, including sugar-sweetened fruit drinks and highly processed sweet and salty snacks, as reasons for not following recommendations. Concerns about food waste, including spoilage and their child refusing to eat some foods (especially vegetables), also increased the perceived cost of serving fruits and vegetables. Discussions about convenience often included mentions of the difficulty of providing fruits and vegetables while on-the-go and contrasted snacks served at home with those served outside the home. Moreover, the widespread availability of sweet and salty pre-packaged snacks, together with the higher cost of healthier packaged snacks, served as barriers to serving healthier snacks while on-the-go. 

Some parents described practical strategies for making fruits and vegetables more convenient snack options, such as buying pre-cut fruit—“*I even buy the sliced apples that are already sliced, too. So you can just put them in the bag and give it to them”*—and packing them for their child to eat on-the-go: *“I put the snacks always in the bag-- a little bag for her. Like grapes, strawberries, carrots. The little baby carrots. And oranges in little pieces”.* Adding water to juice was another common strategy for encouraging their toddler to drink more water while addressing their preferences for sweet drinks: *“Then when I do give him juice, I always put water in it, and he doesn’t seem to know the difference”.*

## 4. Discussion

This qualitative study identified many potential reasons for the low adherence to expert recommendations about providing both healthy snacks and drinks for toddlers, including limitations in caregivers’ understanding of the recommendations and perceived barriers that made it difficult to increase their toddlers’ consumption of recommended options and reduce their consumption of non-recommended ones. These findings contribute to the toddler feeding literature by expanding upon previous studies that examined common reasons why caregivers serve sugar-sweetened beverages to infants, toddlers and young children despite their familiarity with expert recommendations [10,11,12,13,14,16,17,18]. This study demonstrates that many caregivers perceive similar barriers to following healthy snack recommendations, including providing a variety of fruits and vegetables as snacks and avoiding sweet and salty snacks. 

Most participants in this study indicated that they had heard the recommendations about both healthy drinks and snacks for toddlers, but the additional information presented in the concept sheets, including images of non-recommended branded products, elucidated some common misperceptions about these products. As shown in previous studies, many caregivers were not aware that experts consider all drinks with added sugar, including fruit-flavored drinks, to be sugary drinks that should not be served to young children [13,17,18]. In our study, caregivers similarly expressed surprise that many of the snacks that they served and that are widely marketed as appropriate options for toddlers and young children, such as goldfish crackers, graham crackers and “toddler” snacks [31], were included on the concept sheets as examples of sweet and salty snacks that should be avoided. It appeared that caregivers’ perceptions of what qualifies as a healthy choice may also be greatly influenced by the food and drinks served in childcare settings. For example, many assumed that since their childcare provider served foods such as goldfish crackers or graham crackers for snacks, they must be recommended for toddlers.

However, the most frequently cited reasons for not following recommendations emerged when the moderator asked participants, “Do you think this is realistic”? The overwhelming response to this question was “no”, especially in reference to serving only plain water or milk to drink and to consistently providing fruits and vegetables as snacks. The most common barriers to serving both healthier drinks and snacks to toddlers focused on perceptions of their child’s strong preferences for sugary drinks over plain water and milk and for sweet and salty snacks over fruits and vegetables. Many caregivers appeared to believe that these preferences were innate to the child (e.g., he is a “picky eater”) and extremely difficult to change once established, although many recognized that the modeling of consumption behaviors in their home and the provision of non-recommended foods to their child by others (including other family members and in settings outside of the home) played a major role. 

As found in previous studies of sugar-sweetened drinks [10,11,12,13,14,16], many caregivers indicated that family members provided and modeled the consumption of non-recommended snacks and drinks to their toddler, often against their own wishes. Many believed that these practices made it more challenging to get their child to eat or drink healthy options but appeared to feel that they had little control over what other family members (including spouses, grandparents and older siblings) consumed and/or provided to their child. Caregivers’ perceptions of what their child will eat, as well as their beliefs about what products are healthier options for young children, also appear to be greatly influenced by the food and drinks served in childcare settings. Some indicated that they learned about the healthier foods their child would consume when they were provided in childcare.

Practical considerations, including the lower cost and greater convenience and availability of less healthy options, present additional barriers to following expert recommendations. These barriers have been shown in previous studies of sugar-sweetened drinks [11,12,13,14,17,18]. Similar practical considerations also presented barriers to following recommendations to serve a variety of fruits and vegetables as snacks. Pre-packaged and processed sweet and salty snacks appeared to fill caregivers’ need for convenience, especially while on-the-go, which is consistent with a previous study that found that pre-packaged sugar-sweetened beverages are convenient for trips and school lunches [11]. This is also consistent with research showing that toddlers are more likely to consume fruits and vegetables at home while sitting at the table [21]. 

The participants in the focus groups held at the WIC office also questioned the healthy drink recommendations, which they perceived to conflict with advice they received from pediatricians and other trusted sources. This finding is consistent with previous research showing that WIC participants may question their WIC counselor’s advice and that it can conflict with advice from their pediatrician [19,32].

### 4.1. Implications for Nutrition Education and Research

These findings suggest potential intervention targets for improving the nutrition quality of the drinks and snacks that caregivers provide to toddlers. The nutrition education programs utilized by the participants in these groups, including through WIC and family resource centers, appeared to effectively convey general guidance about serving toddlers plain water and milk to drink and a variety of fruits and vegetables for snacks. However, our discussions highlighted the need to address common misperceptions about specific products that are not recommended for toddlers in nutrition education, such as explaining that some popular sugar-sweetened drinks (including fruit drinks) and popular children’s snack foods are not nutritious options for toddlers. Moreover, nutrition education programs should also provide practical advice to help caregivers address the specific barriers identified, including how to communicate with other family members their wishes that their child should not consume certain drinks and snacks and how to reduce the consumption of these products by older siblings and spouses. One randomized controlled trial found that an authoritative food parenting intervention was successful at reducing the consumption of calories from saturated fat and added sugar among mothers of preschoolers [33]. Tips for convenient and low-cost options for serving fruits and vegetables and healthy drinks, including on-the-go, would also be helpful. The caregivers appeared to be looking for practical information to help them navigate the real-life challenges they face when attempting to follow dietary recommendations, rather than strict guidelines that they consider to be unattainable.

Childcare providers also influence what parents feed their toddlers. Thus, initiatives to improve the nutritional quality of foods served in childcare settings, such as the Child and Adult Care Food Program [34], and interventions focused on improving the nutritional quality of foods served in playgroups present an important opportunity. It is likely that childcare providers also experience many of the same barriers when deciding what drinks and snacks to serve the children they care for. These findings also demonstrate the need to develop nutrition education materials, identify interventions and conduct additional research focusing on caregivers’ frequent provision of highly processed sweet and salty snacks to young children in order to complement existing programs for reducing sugary drink consumption. 

### 4.2. Limitations

This study provides additional insights into the reasons behind the reported low adherence with healthy drink and snack recommendations for toddlers identified in a previous quantitative study with a similar caregiver population [26]. However, it has some limitations. All groups were held in one low-income community in Connecticut with a convenience sample, and the results may not be generalizable to other geographic locations and caregivers. The inclusion of WIC participants indicates the applicability to a lower-income population, although we did not assess individual participants’ income. Moreover, this qualitative research was exploratory and focused on the concept sheets provided; thus, the discussion was directed towards identifying barriers to following recommendations and was not entirely open-ended. However, many of the barriers that caregivers described were consistent with other studies that primarily examined barriers to serving healthy drinks among low-income caregivers in a variety of geographic locations, indicating that low-income caregivers may face similar barriers to following both healthy snack and drink recommendations. Focusing the discussion on expert recommendations also posed a potential self-presentation bias in participants’ self-reported knowledge and behaviors, which may have led to the over-reporting of previous knowledge about the recommendations. However, few participants indicated that they followed the recommendations or believed they were realistic, so this bias likely did not greatly limit the discussion of potential barriers. Another limitation is the small number of participants in some focus groups, which may have resulted in a smaller range of experiences in each group and a small number of mentions of some barriers. However, most participants appeared to relate to and were engaged in discussing toddler feeding issues. In addition, the small group sizes may have facilitated participation and in-depth insights into a potentially sensitive topic, as data saturation was achieved, with no additional information about potential barriers discovered in the final groups. Finally, we did not discuss all types of problematic snack foods in the groups. For example, some companies market “organic” and “non-GMO” packaged snacks as healthy options for toddlers. Nutrition education programs aimed at toddler caregivers should also include information to address these types of misleading claims and provide suggestions for healthier and lower-cost homemade options. 

## 5. Conclusions

Providing nutritious drinks and snacks to toddlers and limiting the provision of sugary drinks and processed sweet and salty snacks are critical for the development of life-long healthy food preferences [1]. These focus groups highlight the need for nutrition education to assist caregivers in identifying recommended snack and drink options and providing practical strategies for addressing common barriers to increase the provision of healthier versus unhealthy options, as well as an opportunity to design nutrition education initiatives for encouraging nutritious snack as well as drink provision. These findings also suggest that enlisting childcare providers, pediatricians and other providers in communicating and reinforcing expert recommendations would support caregivers and benefit their young children.

## Figures and Tables

**Table 1:** Individual code definitions and the application to themes/sub-themes describing barriers to following expert recommendations.

Codes	Description	Main Theme/*Sub-Theme* ^1^
Questions about non-recommended products ^2^	Statements indicating surprise or confusion that specific products were included on the lists of non-recommended products.	Understanding/*Misperceptions of non-recommended products*
Other sources of information ^2^	Statements about obtaining different information from different sources (i.e., conflicting information). This also includes specific information or sources of information they have received that they do not trust or believe or that they find confusing.	Understanding*/Questioning recommendations*
Family members ^3^	Statements about how family members influence what the child eats/likes (including foods served); this also includes what older kids/other family members eat and preparing different meals for different people. Comments about how what they eat themselves influences their child are also included.	Child preferences/*Modeling [AND] Others serving*
Setting/context ^3^	Statements about what is served at home vs. other settings; this also includes statements about what is served to their child in other places (e.g., childcare, parties) and how their child’s eating behaviors are different in different settings (e.g., what they eat at home vs. at school).	Child preferences/*Others serving*
Cost of food ^3^	Statements about the cost of foods and beverages (cheap and expensive); this includes all references to price, cost, money or free.	Practical considerations/*Cost*
Food waste ^3^	Statements about throwing away food, food going bad or food not being eaten.	Practical considerations*/Cost* [AND] Child preferences/*Innate*
Convenience and availability ^3^	Statements about selecting a food because it is easy or not easy to serve/prepare/take along; this also includes statements about accessibility (e.g., what is available), messes and scheduling.	Practical considerations/*Convenience*
Eating restrictions ^3^	Includes any references to food allergies, intolerance, health issues that affect one’s diet and special diets (e.g., vegetarian).	Not reported ^4^
Celebrations/special occasions ^3^	Statements about foods for parties, holidays and other special occasions. Only includes mentions of treats if they were for a special occasion.	Not reported ^4^
Marketing ^3^	Statements about information from advertising, messages on product packages (including claims, child features) and other types of marketing. This includes information on company websites, free samples, coupons, etc.	Not reported ^4^
Culture ^3^	Statements about food and feeding that are specific to their culture or country of origin.	Not reported ^4^

^1^ Sub-themes indicated in italics. ^2^ Responses to the moderator question: “*Is this something you’ve heard before? Do you have any questions about what this means”?* ^3^ Responses to the moderator questions: “*Do you think this is realistic? Is it something you think you could do”?* and “*What would make it hard for you to give your child only plain water and milk to drink/ fruits and vegetables for snacks”?* ^4^ Coded but not reported due to a low incidence (i.e., fewer than 10 mentions) and it not being connected to the main themes/sub-themes.

**Table 2:** Representative quotes for the main themes and sub-themes in the thematic analysis.

Themes/Sub-Themes	Healthy Drinks	Healthy Snacks
**Understanding of expert recommendations**		
Misperceptions about non-recommended products	*I didn’t even know Vitamin Water was* [a sugary drink]—*I mean, I know, obviously, it had sugar in it, but I didn’t know it was unhealthy for you.* (WIC group) *I think the Juicy Juice has natural sugars. So that’s supposed to be OK, I think. I don’t know that for a fact.* (WIC group)	*And I’m like-- and I thought granola bars were healthy, too. I don’t give them the chocolate chip ones. I give them the fruit-- with the fruit inside, the soft ones.* (WIC group) *I always thought goldfish was healthy because they serve it at the play group.* (WIC group) *The school gives me a bunch of the little graham crackers. So I thought they were* [healthy]. (WIC group) *I felt like it’s much healthier if I get her a baby cookie versus a chocolate chip cookie from Chips Ahoy. So I just think that they generate healthier stuff for babies, because that’s what the pediatricians want them to do. So yeah, I do. I try to give her baby stuff.*
Questioning recommendations	*With my 1-year-old, the pediatrician was like, he doesn’t need anything more than 8 ounces of milk a day. And at the WIC office was like, no. You still need at least 16 ounces of milk.* *Almond milk it is healthier, they say. So why-- --should give a kid --that kind of milk *[i.e., cow’s milk]? (WIC group) *But, I mean, how can you only give them 4 ounces of juice? I feel like it’s a little bit of torture. They just look at me like, are you serious? And they get so like-- and when I give them water -- I’ve forced them to drink water.* (WIC group)	
**Children’s unhealthy food preferences**		
Innate preferences	*When she’s eating a meal, she’ll drink water. But if you just give her water, she’s not going to drink the water. I put water in her sippy cup, and she threw it at me. I put juice in there and she drank it.*	*So when you say lots of different fruit and vegetables, that doesn’t work for my son. He likes only 1 fruit-- banana.* *So for her, she loves fruit. Bananas, strawberries, pineapples, anything. But when it comes to vegetables, she won’t do it. She rather run up to those Froot Loops, the puff Cheetos, and then she’ll walk over to the snack cabinet... She knows where the snacks are. All her favorites.* *I’ll try to give it to him* [vegetables], *and then he won’t eat it, and then I end up throwing it out…It’s hard to keep trying because I don’t want to waste things.* (WIC group)
Modeling	*My older daughter, she drinks soda. So sometimes when she’s seen the older one, she would say, I want it, I want it. I say, no. And she starts crying.* *That’s not an issue with my granddaughter. She will drink the milk and the water and not ask for sugary* [i.e., sugary drinks] *because there’s no older kids in the household that she sees drinking them or asking for them*. *I personally drink the Vitamin Water…So they see me. I can’t help it. They want to do what I do.* (WIC group)	*With my youngest daughter, my challenge is trying not to introduce candies or sweets because my older daughter [12-year-old] is sometimes drinking soda or eating candies. So the little one is trying to eat the same.* *He* [my 5-year-old] *will go hungry all day if you do not give him what he wants. And rather than him going all day hungry, I’ll give in and be like, OK. You can have nuggets. You can have fries. But then the 1-year-old sees him eating it and he won’t want to eat his fruits and veggies any more, he wants nuggets and fries*.
Others serving non-recommended products	*Well, it’s kind of realistic *[i.e., drink recommendations]. *You don’t have to buy it, but they’re probably going to get it somewhere.* *Nana goes, here, just have a Sprite or something. And it’s annoying, but I’m just like, I’m not driving myself crazy running around after to see what you’re doing, see what you’re drinking, or whatever. And I tell her, but they don’t care. They support it sometimes, but not all the time.* (WIC group) *But people are willing to give you free sugar-loaded things *[drinks] *for your kids all the time. And sometimes I feel like the bad person that I’m blocking everything. No, no, no. She can’t have that, she can’t have that. Just to try to get her to eat better and not to introduce those foods to her.*	*Before, he loved his veggies and his fruits. But then I think when he goes to Nana’s house and with dad and he gets to eat that junk food, that’s all he wants to eat now, is junk.* *I think the most difficult thing is other people feeding a child whatever they brought, or things like that. Like, you know, you’re home. They’re comfortable. You can lay down the rules. But when they’re in somebody else’s care, sometimes everything goes out the window that day.* *Whatever they want-- He gives them it. Going behind my back. You’re with daddy now, so you can get whatever you want.* *The challenges I do have with her eating is that...when she’s with me, she eats extremely well. However, when she’s being introduced to all these things, whether in daycare, or in camp, or with family…she seems to favor those foods, opposed to the more whole foods.*
**Practical considerations**		
Cost	*There’s something called Hi-C. Those are usually $1.99 for the pack. They’ll last me a while. So I’ll buy that… Whatever’s on sale. The cheapest box, that’s the one I’m getting.* (WIC group)	*I give my kids a lot of fruit, though…I mean, and there’s only so much of it. Once it’s done, I’m like, OK, you guys have these snacks now. Because trying to eat healthy and fresh is really expensive. It’s really expensive… I can’t afford it all the time.* *If I want to buy all these veggies and fruits, that comes up to like $60. And I can only go $200 a week on groceries.* [Describing SNAP benefits] *I still have to get my meats, I still have to get my dairy and my greens, everything.* (WIC group) *And if you do run into Stop and Shop to pick up a little vegetable pre-packaged with the hummus, that’ll cost you $3, $4, $5. Yeah. It’s ridiculous. And you can get a thing of pretzels for 99 cents.*
Convenience/availability		*I always keep cereal in the car… I rarely will have fruit in there because this I can keep in my bag forever.* *Those I do buy* [packaged snacks] *when we are outside, in the moment…But when they are in my house, what they eat is fruits and vegetables.* *When you’re out and you forget to pack your snack, to pick up something quickly, the junk food is more-- it’s more available-- --than the fruit, than the vegetable.* *So, it’s hard to go in the grocery and buy healthy stuff. You never know what to buy. Like, fruity snack, it’s like, oh, it’s fruit. But I know it’s not-- even graham crackers.*

## Data Availability

Data are available upon request.
